# Current Progress on Assessing the Prognosis for Anti-N-Methyl-D-Aspartate Receptor (NMDAR) Encephalitis

**DOI:** 10.1155/2020/7506590

**Published:** 2020-04-14

**Authors:** Hao Wang, Zheng Xiao

**Affiliations:** ^1^Neurology Department, Chongqing Medical University, Chongqing, China; ^2^Neurology Department at the First Affiliated Hospital of Chongqing Medical University, Chongqing, China

## Abstract

Anti-N-methyl-D-aspartate receptor (NMDAR) encephalitis is the most common antineuronal antibody encephalitis in autoimmune encephalitis found at present. It has complex clinical manifestations such as psychiatric and behavioral abnormalities, seizures, movement disorders, consciousness, and autonomic dysfunction. The relationship between those manifestations and prognosis is unclear. Electroencephalography (EEG) is gradually becoming useful in the evaluation of the prognosis of anti-NMDAR encephalitis patients, while imaging and antibody testing have a limited prognostic value. Starting the patients on adequate treatments (such as immunotherapy) in a timely fashion has a positive impact on their prognosis. Nevertheless, research on the prognosis of anti-NMDAR encephalitis remains scarce. Here, we review the current advances of prognosis-related research from the clinical manifestations of the disease and auxiliary examinations such as EEG, magnetic resonance imaging (MRI), 18F fluorodeoxyglucose positron emission tomography (FDG-PET), and antibody measurement. In addition, we also discuss the impact of different treatment options on prognosis. In-depth research on the prognosis of patients with anti-NMDAR encephalitis will contribute to a better understanding of this disease, leading to better treatments options and, ultimately, a better prognosis.

## 1. Introduction

Anti-N-methyl-D-aspartate receptor (NMDAR) encephalitis is an autoimmune disease that is characterized by the presence of neuropsychiatric symptoms. The standard for diagnosis is the detection of anti-NMDAR GluN1 subunit Immunoglobulin G (IgG) antibodies in the cerebrospinal fluid (CSF) [1]. As reviewed in [1], anti-NMDAR IgG antibodies present in the CSF bind to the extracellular N-terminal domain of the NMDAR NR1 subunit, embedded within postsynaptic membranes of central nervous system (CNS) neurons. This leads to the cross-linking and internalization of NMDARs, which reduces the number of NMDARs and NMDAR clusters on the surface of neurons, leading to dysfunction and disease. Anti-NMDAR encephalitis is most commonly diagnosed in children and young people and is often associated with tumors, such as ovarian teratomas, which more frequently occur in women over 18 years of age. In addition, viral infections such as herpes simplex virus can also induce anti-NMDAR encephalitis. Typical clinical manifestations include psychiatric and behavioral abnormalities, seizures, recent memory impairments, involuntary movements, speech disorders, conscious disturbances, and autonomic dysfunction. Currently, symptomatic treatment and immunotherapy (intravenous methylprednisolone (IVMP), intravenous immunoglobulin (IVIG), or plasma exchange (PE), etc.) are available for the treatment of anti-NMDAR encephalitis.

The disease was formally proposed by Dalmau and others in 2008 [1]. Its incidence and overall prognosis are currently unknown, although a Dutch study estimated the incidence to be 2-3 cases per 1 million people [2]. As clinicians gradually understand the disease, an increasing number of patients are diagnosed [3]. The British California Encephalitis Study showed that the incidence of anti-NMDAR encephalitis has exceeded that of any single virus-induced encephalitis. It further showed that anti-NMDAR encephalitis is the most common antineuronal antibody-mediated encephalitis [3]. In the current largest anti-NMDAR encephalitis cohort study published in 2013 [4], it was shown that after 24 months of follow-up, 78% of patients achieved a good prognosis, 13% of patients relapsed, and 5% of patients died. Since then, no other similar large-scale study on anti-NMDAR encephalitis has been conducted. However, in 2017, de Montmollin [5] and others analyzed 77 anti-NMDAR encephalitis patients admitted to the ICU and showed that 77% had a positive prognosis after 2 years of follow-up, while 4% died. Many factors can affect a patient's prognosis. However, both primary research and relevant reviews focusing on the prediction of anti-NMDAR encephalitis are scarce. In this review, we summarize the current progress on understanding and predicting the prognosis for anti-NMDAR encephalitis given its pathogenesis, clinical manifestations, auxiliary inspection, and treatments.

## 2. Pathogenesis

Autoimmune encephalitis (AE) is an inflammatory disease of the CNS which is caused by an abnormal immune response against the body's own neuronal components. It can be divided into paraneoplastic AE and nonparaneoplastic AE, based on whether a tumor is present or not. Paraneoplastic AE can further be divided into intracellular antigen-antibody-associated encephalitis and cell surface antigen-antibody-associated encephalitis based on the location of the specific autoimmunogenic antigens. Intracellular antigen antibody encephalitis includes anti-Hu, anti-Ma2, and anti-GAD antibody encephalitis. Cell surface antigen antibody encephalitides are more common [6] and include anti-NMDAR, anti-leucine-rich glioma inactivating protein 1 (LGI1) antibody-related, and anti-*γ*-aminobutyric acid type B receptor (GABABR) antibody-related brain encephalitis, with the first having the highest incidence.

Anti-NMDAR encephalitis is an autoimmune neuroinflammatory disease mediated by antibodies against the NMDAR GluN1 subunit. In an endogenous rodent model of anti-NMDAR, Linnoila et al. showed that 67% of mice developed serum anti-NMDAR antibodies after infection with herpes simplex virus 1 [7]. In addition to viral infections, tumors and other unknown causes could also lead to autoimmune response against exposed neuronal antigens. In the case of anti-NMDAR encephalitis, exposed GluN1 subunit antigens become the target of autoantibodies against NMDAR [8]. In this process, the immune system first produces memory B cells, which could subsequently be recruited to the brain through the activity of various cytokines, chemokines, and inflammatory mediators. In the brain, memory B cells can be stimulated by antigens and can mature, proliferate, and differentiate into plasma cells, in order to produce antibodies [8–11].

The NMDAR is widely present in the CNS, especially in the frontal cortex, hippocampus, and thalamus. It participates in synaptic transmission and signal transmission between neurons and is related to advanced neuronal functions, including memory, cognition, and behavior. Neurons and animals exposed to patient anti-NMDAR antibodies *in vitro* and *in vivo*, respectively, confirmed that the specific binding of CSF anti-NMDAR antibodies to their cognate receptor does not lead to the death of nerve cells, but rather to the reversible reduction and functional decline of NMDAR [12, 13]. A series of abnormal psychiatric and behavioral movement disorders and other clinical symptoms became evident after the antibody-mediated reduction of NMDAR. Unlike T-cell-mediated anti-Hu antibody encephalitis and other types of intracellular neuronal encephalitis, neuronal death is not observed in anti-NMDAR encephalitis [14]. As such, anti-NMDAR encephalitis has a relatively good prognosis after treatment [1, 15, 16].

## 3. Clinical Manifestations

### 3.1. Psychiatric Symptoms

Psychiatric symptoms are the earliest, most common, and in some cases, the only clinical manifestations of anti-NMDAR encephalitis [17, 18]. They are more common in young and female patients [19, 20]. Common psychiatric symptoms include visual and auditory hallucinations, schizophrenia-like symptoms, anxiety and depression, mania, and abnormal behavior. Because the identification of psychiatric symptoms is difficult, the median admission time for patients presenting mainly with psychiatric symptoms is significantly longer than that of those with neurological dysfunctions, such as epilepsy (14 vs. 2 days, respectively), where neurological dysfunctions are the first manifestations [21]. Consequently, patients presenting mainly with psychiatric symptoms experience a significant delay in the administration of immunotherapy. In turn, this may affect their recovery, especially in terms of psychiatric symptoms and cognitive impairment. Previous studies have mostly focused on positive symptoms, such as delusions and mania. However, Gibson and others have shown that patients with anti-NMDAR encephalitis have obvious negative symptoms, cognitive impairments, and psychiatric disorders that do not match the severity of the symptoms [19]. The clinical manifestations of the negative symptoms can also be explained by the mechanism of antibody-mediated NMDAR downregulation. Indeed, in anti-NMDAR encephalitis, animal models which mimic the clinical course of the disease are characterized by memory loss and lack of pleasure [12]. Cainelli et al. conducted a long-term follow-up study of children diagnosed with anti-NMDAR encephalitis and found that about half of them had long-term attention disorders and executive dysfunction, while some of them had long-term mental behavior abnormalities [22]. Positive symptoms can be almost completely ameliorated after active treatment. By contrast, negative symptoms, including cognitive and memory impairments and thinking disorders, can persist in patients over the long-term and even be permanent, leading to a poor prognosis [23, 24]. In children, since the nervous system is not fully developed at the time of onset, the impact of the disease may be more pronounced [23]. Ameliorating cognitive impairments is of great significance for patients' long-term rehabilitation and for improving their quality of life. However, research on cognitive impairment is still limited, and further large-scale and prospective studies are needed.

### 3.2. Seizure

Seizures are also a core clinical manifestation of anti-NMDAR encephalitis, occurring in about 60-70% of patients [4, 25, 26] and being more common in children and young patients. Before puberty, epilepsy is more common in females than in males. After puberty, the situation is reversed, possibly due to changes in sex hormone levels [4, 25, 27, 28]. Seizure symptoms are diverse and can include typical generalized tonic-clonic seizures, focal seizures (with or without loss of consciousness), a persistent state of epilepsy, and a persistent state of refractory epilepsy. Some patients have 2 or more types of seizures [20, 26]. Epilepsy in patients with anti-NMDAR encephalitis is mediated by an autoimmune mechanism, and most patients have significantly reduced seizures after their encephalitis has been treated. For example, de Bruijn et al. assessed whether seizures could be ameliorated in patients with anti-NMDAR encephalitis who experienced seizures throughout the courses of their disease [29]. Most patients saw an improvement as a result of immunotherapy alone. For some, the addition of antiepileptic drugs after the immunotherapy was necessary to eliminate seizures [29]. In another study where epilepsy occurred in 81% of 109 patients, a single-agent antiepileptic response eliminated seizures for up to 2-weeks after the administrations. Moreover, all patients had no apparent seizures after 2 years of follow-up [28]. de Bruijn et al. suggested that epilepsy is a clinical symptom of anti-NMDAR encephalitis in a particular stage and can be gradually eliminated with the use of immunotherapy combined with the use of antiepileptic drugs [29]. This view is in line with the results of Taraschenko et al. [14]. Their group has recently shown a significant increase in seizures in mice exposed to anti-NMDAR antibodies, hinting that autoantibodies *per se* have the potential to induce seizures. Therefore, some believe that epilepsy or epilepsy status does not affect the prognosis of patients [30]. Although patients with anti-NMDAR encephalitis have epileptic symptoms, they should not be immediately diagnosed with epilepsy. However, if they are diagnosed with epilepsy or need to take antiepileptic drugs for prolonged periods, patients should be followed up for at least 1 year. It is further recommended that antiepileptic treatments are phased out during the recovery period [26, 28].

### 3.3. Movement Disorders

Abnormal movements are another common manifestation of anti-NMDAR encephalitis and mainly present as facial dyskinesia or involuntary movements of the limbs. In addition, patients can present with complex clinical manifestations, such as myotonia, dystonia, bradykinesia, and eyelid spasms. About 75% of adults and 95% of children develop movement disorders [1, 4, 31]. Some scholars believe that abnormal psychiatric and behavior symptoms and seizures are early clinical manifestations after the onset of anti-NMDAR encephalitis, which may last, in average, 10 to 20 days [26, 32]. The presence of movement disorders indicates that the disease has progressed to a more advanced stage. To explain this theory, it is postulated that early in the disease, serum antibodies act on the gray matter of the cerebral cortex to produce a series of clinical symptoms. As the disease progresses, intrathecally synthesized antibodies are produced and are able to act on the subcortical structure to produce additional clinical symptoms. Studies have shown the interval between different stages to be about 18.6 days [32, 33]. As a result of the facial dyskinesia, some patients may suffer from secondary local organ damage, such as to the oral cavity and tongue. Symptomatic treatment can improve the symptoms of patients. Nevertheless, the focus of treatment should still be immunotherapy. After active treatment, the symptoms are mostly ameliorated and there are no obvious sequelae. No studies have confirmed the association between movement disorders and poor prognosis.

### 3.4. Autonomic Dysfunction

Common autonomic dysfunctions caused by anti-NMDAR encephalitis include tachycardia, bradycardia, arrhythmia, cardiac arrest, diarrhea, central hypoventilation, and excessive ventilation [34, 35]. Autonomic dysfunctions can directly affect the patient's prognosis and long-term recovery. Symptoms such as arrhythmia, cardiac arrest, and central hypoventilation may be the main reasons for ICU support or death. Some patients with cardiac arrest may need a pacemaker. Heartbeat activity is regulated by cardiac sympathetic and parasympathetic nerves. Animal studies have shown that multiple regions of the medial cerebrum including the island lobe, cingulate gyrus, and amygdala are involved in the regulation of cardiac sympathetic and parasympathetic nerves. Consequently, intracranial lesions may affect the neuromodulation of the heart, leading to arrhythmia and cardiac arrest [35]. Simultaneously, anti-NMDAR encephalitis-related epileptic activity may induce synchronous firing of the autonomic nerves of the heart, leading to lethal arrhythmias [36, 37]. In a study with anti-NMDAR encephalitis patients, Wang et al. [20] showed that 28% of patients had hypoventilation, 26% required mechanical ventilation, and 20% had a tracheotomy and required ICU support. Moreover, studies have shown that ICU treatment can be used an independent risk factor for death [38]. Schubert [34] and others have pointed out that the presence of autonomic nervous dysfunction during hospitalization and the use of mechanical ventilation were significantly associated with poor neurological function at discharge and may be associated with poor long-term prognosis [39]. Therefore, the emergence of autonomic nerve dysfunction during the course of disease can lead to other serious complications and affect the prognosis of patients. The relationship between autonomic nerve dysfunction and poor prognosis requires further study.

The clinical manifestations of anti-NMDAR encephalitis are complex. Symptoms such as epilepsy and movement disorders can gradually disappear with treatment and have little correlation with prognosis. However, patients may have symptoms, such as cognitive impairment, which have a slow recovery process and may persist long-term, thereby impacting the patients' quality of life. Autonomic dysfunction is strongly related to prognosis, which may lead to serious complications and poor prognosis, which requires active intervention.

## 4. Auxiliary Inspection

### 4.1. EEG

In the diagnosis, treatment, and evaluation of anti-NMDAR encephalitis, EEG plays an important role due to its convenience, speed, and noninvasiveness. Seizures and movement disorders in anti-NMDAR encephalitis are easily confused [40], and EEG has been posited as a necessary auxiliary test for the differential diagnosis [41]. Studies have shown that in the most severe cases of anti-NMDAR encephalitis, EEG abnormalities are more helpful for diagnosing the disease than magnetic resonance imaging (MRI) (98.4% vs. 46.8%) [42]. In addition to seizures, common EEG abnormalities in anti-NMDAR encephalitis include nonspecific diffuse slow waves [43] and specific delta brushes (extreme delta brush (EDB)), among others [41]. Since EDB is more common in comatose patients and other severe cases, it is currently considered to indicate a poor prognosis [21, 44, 45]. However, some studies suggest that EDB is of limited significance in predicting patient prognosis; so with respect to anti-NMDAR encephalitis, more research is needed to understand the relationship between EDB and prognosis [42]. Besides, Zhang and others have shown that patients with a normal EEG background, epileptic discharge, polymorphic delta rhythm, and diffuse beta activity of EEG have a good long-term prognosis [42].

### 4.2. Imaging

Imaging findings in anti-NMDAR encephalitis are complex, show poor specificity, and have limited prognostic value. Common imaging tools used for the diagnosis of anti-NMDAR encephalitis include MRI and FDG-PET. The incidence of abnormal MRI findings in cases of anti-NMDAR varies from 11% to 83% [18, 46]. Indeed, a systematic review of 56 clinical studies showed that less than 50% of patients had abnormal MRI findings [47], with most abnormalities coming from T2-weighted scans as well as a high FLAIR signal. Abnormal findings are most commonly found in the temporal lobe, in addition to the cerebral cortex and subcortical white matter regions. The frontal lobe, hippocampus, and cerebellum can also be affected. However, Gabilondo et al. [48] suggested that abnormal MRI manifestations were not associated with anti-NMDAR encephalitis recurrence. In addition to abnormal MRI findings, some patients can show reversible diffuse brain atrophy (DCA) and even brain atrophy, including progressive cerebellar atrophy. Studies have shown that DCA patients require more mechanical ventilation and have longer hospital stays [49]. However, in a clinical study with a median follow-up of 68 months, 33% of patients with DCA and some brain atrophy patients showed strong recoveries. Nevertheless, progressive cerebellar atrophy was irreversible, and in this study, cerebellar atrophy was strongly associated with a poor prognosis [49]. By using multimodal MRI, Finke et al. found that the hippocampal connectivity and fractional anisotropy in the white matter of anti-NMDAR encephalitis patients were reduced, mainly the cingulate gyrus. Importantly, these changes were not detected when using conventional MRI [50]. The abovementioned pathological changes may be related to the patient's cognitive impairment and severity of the disease, which may affect the prognosis.

Compared with lower-sensitivity MRI, FDG-PET is extremely sensitive for detecting the most severe stages of anti-NMDAR encephalitis [47, 51]. Typically, anti-NMDAR encephalitis is characterized by hyperfrontal and temporal lobe metabolism and a decrease in parietal and occipital lobe metabolism. The recovery period is mainly characterized by diffuse cortical metabolism [52]. The metabolic performance, measured by PET-computed tomography (CT), is almost normal in patients with negative antibody recovery and no obvious clinical symptoms. However, it may appear abnormal again as the patient relapses. Thus, PET-CT can be used to assist in the diagnosis of anti-NMDAR encephalitis, helping to determine the degree of disease progression based on the specific metabolic pattern of disease changes. However, further research is needed to guide long-term prognosis assessments.

### 4.3. Laboratory Tests

At present, the detection of IgG antibodies against the NMDAR GluN1 subunit in the CSF is the main diagnostic method for anti-NMDAR encephalitis. Studies have shown that the CSF antibody titer is maximal when the disease is most severe. If the disease is controlled, the CSF antibody titer gradually decreases with time, although it may remain positive for a long time [53]. Gresa-Arribas and others have shown that patients with poor outcomes had higher levels of CSF and serum anti-NMDAR antibodies than patients with positive outcomes [53]. Likewise, the concentration of CSF antibodies increased when encephalitis recurred. This conclusion is also supported by the findings of Schneider et al. [45], given that intrathecally synthesized antibodies can be retained for many years after the clinical symptoms have disappeared. Therefore, even though antibody titers are related to the onset of the disease, the improvement of symptoms is not associated with a decline in antibody titers [5, 30]. Consequently, Dalmau and others believe that clinical evaluation, rather than antibody levels, should be the main decision-making tool to guide treatment [26]. The relationship between CSF antibody titer, prognosis, and relapse is not clear and warrants further research.

## 5. Treatment

### 5.1. Immunotherapy

There is no uniform standard immunotherapy for anti-NMDAR encephalitis. At present, immunomodulatory therapy is mainly used. Immunomodulatory drugs can be divided into first-line and second-line. First-line immunotherapy drugs include intravenous methylprednisolone (IVMP), intravenous immunoglobulin (IVIG), or PE. High-dose methylprednisolone therapy can regulate T lymphocyte function and reduce inflammatory responses [54]; it is currently the most commonly used immunotherapy and is given as an intravenous infusion of 30 mg/(Kg·d), which is gradually reduced during the course of the treatment [55]. IVIG can inhibit humoral and cellular immunity and regulate immune responses through a variety of mechanisms [56]. Commonly, IVIG is given at a dosage of 0.4 g/Kg for 5 days, and it can be reused later according to the condition of the disease [57]. PE can reduce the CSF anti-NMDAR antibody titer by removing these antibodies from the blood, thereby ameliorating the disease [57]. In the course of immunotherapy, IVMP, IVIG, and PE therapy can be used in combination, depending on the patient's condition. However, currently, the order of drug administration and choice of protocols are controversial [57, 58]. Second-line immunotherapy drugs include rituximab, cyclophosphamide, azathioprine, and mycophenolate mofetil [26]. Studies have shown that second-line immunotherapies can be started in patients who have failed to respond to first-line immunotherapies. In those cases, second-line immunotherapies have improved the prognosis of patients when compared to those who did not have any further form of immunotherapy [4]. Moreover, patients who were started on second-line immunotherapy without receiving any first-line immunotherapy drug had a better prognosis than untreated patients [4]. For patients who did not improve after first-line and second-line treatments, clinical trials are currently testing the effectiveness of intrathecal injections of methotrexate and glucocorticoids as an attempt to block the intrathecal synthesis of anti-NMDAR antibodies [59]. So far, this treatment showed promise by improving the symptoms of patients and reducing CSF antibody titers.

Current research suggests that early initiation of immunotherapy can significantly improve the prognosis of patients and reduce the recurrence rate [4, 48]. Studies on second-line treatment have shown that rituximab can significantly improve the prognosis of patients [60]. By contrast, some research has indicated that starting immunotherapy as soon as possible does not reduce mortality of anti-NMDAR encephalitis patients [38]. However, some researchers have proposed that third-line treatments should be used if second-line treatments fail. Bortezomib and tocilizumab are examples of third-line treatments; however, the effectiveness of bortezomib remains controversial [26]. Those who continued to be treated with tocilizumab after initial treatment failure may have shown a better prognosis at follow-up after 24 months [61]. Although most current studies on the prognosis of anti-NDMAR encephalitis demonstrate immunotherapy to be efficacious and able to gradually restore patients to baseline levels, current research has gradually progressed toward addressing patients who have not fully recovered [62]. More research is needed to understand the sequelae following immunotherapy in anti-NDMAR encephalitis patients.

### 5.2. Tumor Treatment

Anti-NMDAR encephalitis is often associated with tumors. Patients who present with tumors also need to undergo removal of the tumor. The occurrence of tumors depends on age, sex, and ethnicity. Female patients mostly develop ovarian tumors, and patients over 12 years of age have a 52% chance of developing a teratoma [4]. The younger the patient, the less likely they are to have tumors [63]. Most tumors in men are germ cell tumors, and only 5% of male anti-NMDAR encephalitis patients over 18 years of age have underlying tumors [63]. In addition, liver neuroendocrine tumors, uterine neuroendocrine tumors, small cell lung cancer, and cancers of unknown origin have recently been associated to anti-NDMAR encephalitis [62]. In these cases, tumor resection in conjunction with simultaneous immunotherapy can significantly accelerate the amelioration of the disease and reduce the need for second-line treatments [63]. In a 2011 study by Dalmau et al., 80% of tumor patients showed a substantial improvement after a combination of tumor resection and first-line immunotherapy. By contrast, only 48% of tumor-free patients showed similar improvements after first-line immunotherapy. Some studies suggest that patients without tumors show more significant cognitive deficits. However, in assessing the long-term prognosis, follow-up results show that patients with and without tumors are statistically indistinguishable and both will eventually show a substantial improvement in symptoms [63]. However, patients without tumors who do not receive (or are delayed in receiving) immunotherapy may have a poor prognosis [31]. A 2013 study by Titulaer and others also demonstrated that there was no obvious correlation between the presence of tumors and long-term prognosis [4]. Moreover, they found that patients who had their tumors resected had a lower recurrence rate for anti-NMDAR encephalitis during follow-up. Similarly, in a cohort study of severe anti-NMDAR encephalitis patients who required ICU treatment, the presence of a tumor did not affect patient prognosis [5]. Indeed, a systematic analysis by Broadley et al. [30] in 2019 supports that anti-NMDAR encephalitis patients with an underlying tumor may have shorter recovery times and lower recurrence rates.

### 5.3. ICU Admission

A subset of anti-NMDAR encephalitis patients can display severe clinical symptoms with complications such as cognitive impairment, unconsciousness, status epilepticus, hypoventilation, and cardiac arrest. In this aspect, the ICU can perform a number of supportive treatments to relieve the condition. While there is a necessity for ICU treatments, the long-term prognosis for patients admitted to the ICU remains inconclusive. For example, in a 2013 clinical study involving 577 patients with anti-NMDAR encephalitis, Titulaer et al. showed that in univariate and multivariate analyses, not requiring ICU support in 4 weeks after admission was significantly associated with favorable patient outcomes [4]. de Montmollin et al. showed that, across 77 anti-NMDAR encephalitis patients from 52 ICU treatment centers, even if ICU treatment was required, the prognosis remained satisfactory [5]. They further affirmed that the early initiation of immunotherapy was beneficial and is an independent factor of a good prognosis [5]. However, when analyzing the cause of death of 96 patients with anti-NMDAR encephalitis, Chi and Zhou showed that the mortality rate was as high as 11.46%, and significantly associated with continued ICU treatment [38]. Broadley et al. conducted a systematic review and confirmed that on-admission ICU support treatment was necessary and could impact the long-term prognosis of anti-NMDAR encephalitis patients [30]. Therefore, patients who require ICU support treatment to address severe manifestations of anti-NMDAR encephalitis have a significantly higher risk for a poor prognosis and death. Moreover, the overall prognosis of the disease is significantly related to a timely administration of immunotherapy. Importantly, patients who have needed ICU support can still achieve a good prognosis. Thus, in the ICU, a multidisciplinary and comprehensive treatment strategy based on the patient's condition can significantly improve the patient's prognosis [45].

## 6. Conclusions

The clinical manifestations of anti-NMDAR encephalitis are complicated. Patients tend to exhibit severe symptoms, and recovery is slow. At present, research on the prognosis of anti-NMDAR encephalitis remains limited. We summarized the relevant research on the anti-NMDAR encephalitis, with a focus on factors affecting prognosis, such as disease pathogenesis, clinical manifestations, auxiliary examination, and treatments. Autonomic dysfunction and related complications may affect long-term prognosis, with cognitive impairment being the main dysfunction encountered during the long-term recovery of patients with anti-NMDAR encephalitis. When the abovementioned clinical manifestations appear, they should be actively treated as soon as possible in order to improve the chances for a positive outcome. Auxiliary inspection has gradually shown a capacity to predict prognosis. The value of EEG for predicting the long-term prognosis of patients is gradually highlighting its value, but the relationship between CSF antibodies, imaging, and prognosis needs further exploration. Immunotherapy started at an early stage is still the best treatment plan to obtain a good prognosis, but for some patients with poor curative effects, more and more effective treatment methods need to be explored to shorten the clinical course of patients, reduce disease complications and sequelae, and obtain a better prognosis. At present, there are still a limited number of studies focusing on the prognosis of anti-NMDAR encephalitis. Our review showcases future research directions. Clinical manifestations, auxiliary examinations, treatment options, and complications may all become prognostic indicators for patients, and we look forward to further prospective research in the future that is aimed at overcoming these difficulties.
